# The Effect of Aerobic Exercise on Intrahepatocellular and Intramyocellular Lipids in Healthy Subjects

**DOI:** 10.1371/journal.pone.0070865

**Published:** 2013-08-14

**Authors:** Andrea Egger, Roland Kreis, Sabin Allemann, Christoph Stettler, Peter Diem, Tania Buehler, Chris Boesch, Emanuel R. Christ

**Affiliations:** 1 Division of Endocrinology, Diabetology and Clinical Nutrition, University Hospital of Bern, Inselspital, Bern, Switzerland; 2 Department of Clinical Research, Magnetic Resonance Spectroscopy and Methodology, University of Bern, Bern, Switzerland; University of Milan, Italy

## Abstract

**Background:**

Intrahepatocellular (IHCL) and intramyocellular (IMCL) lipids are ectopic lipid stores. Aerobic exercise results in IMCL utilization in subjects over a broad range of exercise capacity. IMCL and IHCL have been related to impaired insulin action at the skeletal muscle and hepatic level, respectively. The acute effect of aerobic exercise on IHCL is unknown. Possible regulatory factors include exercise capacity, insulin sensitivity and fat availability subcutaneous and visceral fat mass).

**Aim:**

To concomitantly investigate the effect of aerobic exercise on IHCL and IMCL in healthy subjects, using Magnetic Resonance spectroscopy.

**Methods:**

Normal weight, healthy subjects were included. Visit 1 consisted of a determination of VO_2max_ on a treadmill. Visit 2 comprised the assessment of hepatic and peripheral insulin sensitivity by a two-step hyperinsulinaemic euglycaemic clamp. At Visit 3, subcutaneous and visceral fat mass were assessed by whole body MRI, IHCL and IMCL before and after a 2-hours aerobic exercise (50% of VO_2max_) using ^1^H-MR-spectroscopy.

**Results:**

Eighteen volunteers (12M, 6F) were enrolled in the study (age, 37.6±3.2 years, mean±SEM; VO_2max_, 53.4±2.9 mL/kg/min). Two hours aerobic exercise resulted in a significant decrease in IMCL (−22.6±3.3, % from baseline) and increase in IHCL (+34.9±7.6, % from baseline). There was no significant correlation between the exercise-induced changes in IMCL and IHCL and exercise capacity, subcutaneous and visceral fat mass and hepatic or peripheral insulin sensitivity.

**Conclusions:**

IMCL and IHCL are flexible ectopic lipid stores that are acutely influenced by physical exercise, albeit in different directions.

**Trial Registration:**

ClinicalTrial.gov NCT00491582

## Introduction

Intramyocellular (IMCL) and intrahepatocellular (IHCL) lipids are so called ectopic lipid depots. IMCL have been shown to significantly contribute to fuel metabolism in physically active healthy subjects during exercise [1]. On the other hand, it is believed that ectopic lipids and in particular their degradation products impair insulin action in the respective tissues [2], [3] . There is substantial data indicating that life style intervention (exercise and diet) significantly influences ectopic lipids [2]. Using Magnetic Resonance spectroscopy (^1^H-MR-spectroscopy), these depots can be repeatedly assessed in a non-invasive manner [4].

We and others [5]–[11] have demonstrated that IMCL are flexible lipid stores that can be repleted by standardized dietary fat intake and depleted by defined physical exercise over a broad range of exercise capacity and insulin sensitivity [6], [8], [10], [12]. Interestingly, high IMCL-concentrations can be found in physically active healthy subjects (i.e. insulin sensitive subjects) and in insulin resistant subjects [8], [11]. Using a dietary protocol including fat snacks combined with physical exercise it has been shown that healthy subjects are capable to increase their intramyocellular lipids by dietary fat intake and use these energy stores by physical exercise [8], [9], [11]. Importantly, sedentary healthy subjects and insulin resistant patients exhibit a decreased capacity to deplete their IMCL stores by physical exercise [6], [13]. These findings suggest that the ability to deplete IMCL by physical exercise may be related to exercise capacity. Results of studies using ^1^H-MR-spectroscopy indicate that the ability to deplete IMCL during exercise is associated with quantitative and qualitative changes in number and function of mitochondria [3], [5].

**Figure 1:**
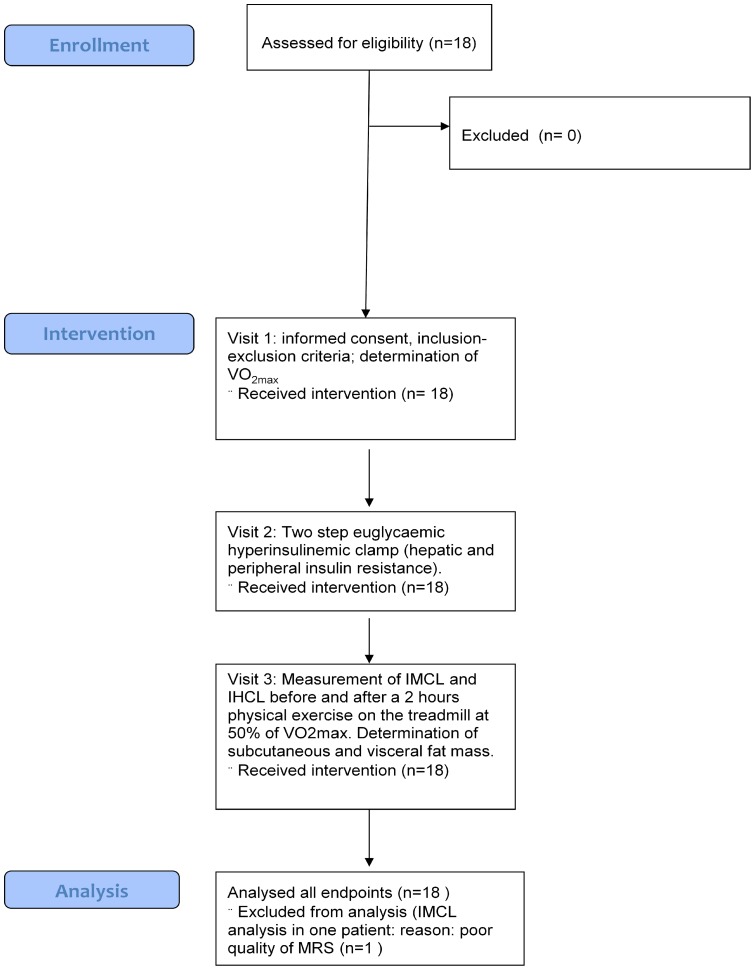
Flow sheet of patients, intervention and analysis. Flow sheet of enrolled patients, intervention and analysis of data. IMCL  =  intramyocellular lipids.

IHCL have been demonstrated to be linked to markers of insulin resistance [14]–[17]. In addition, several data suggest that fat mass, in particular visceral fat mass, is positively correlated with IHCL [14]–[17] suggesting that the availability of fat (fat mass and circulatory free fatty acids; FFA) may be involved in mediating the impaired insulin action at the hepatic level [18]. Regular physical activity over 12 weeks in adults [19], [20] and adolescent boys [21] without caloric restriction has shown that IHCL decreased in parallel with a decrease in visceral fat mass and an improvement in insulin sensitivity. Interestingly, in adolescent boys particularly resistance exercise appears to significantly impact on insulin sensitivity whereas the influence of aerobic exercise or resistance training on IHCL and visceral fat mass is comparable [21].

**Figure 2:**
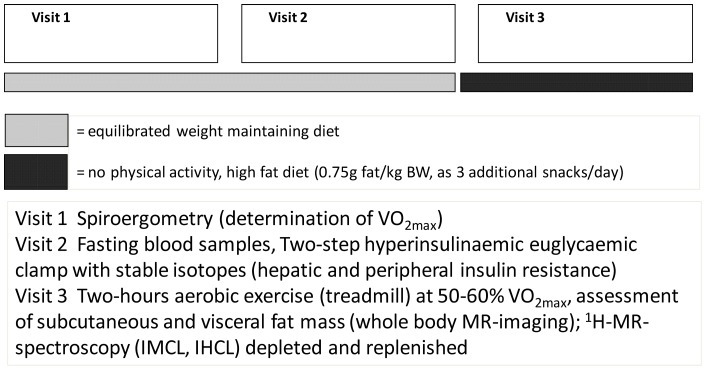
Study protocol. The clinical protocol included three visits and two periods. Period 1: weight maintaining diet, period 2: no physical activity, high fat diet (as additional fat snacks, 0.75 g fat/kgBW for 1.5 days prior to visit 3). Visit 1 Spiroergometry (calculation of 50% VO_2max_, trial on the treadmill). Visit 2 Two-step hyperinsulinaemic euglycaemic clamp using stable isotope technique. Visit 3 Assessment of intramyocellular (IMCL) and intrahepatocellular lipids (IHCL) before and after two hours aerobic exercise at 50% VO_2max_. Measurements of subcutaneous and visceral fat mass using MRI imaging.

So far, ^1^H-MR-spectroscopy has not been used to assess whether physical activity acutely induces sizeable changes in IHCL. On the other hand it is established that a demand for fat as energy substrate typically occurs in response to physical activity [22], [23]. The triglyceride fatty acid cycle provides FFA as substrate by hydrolyzing triglycerides from adipose tissues and releasing them into the circulation [22], [23]. They are – according to the energetic needs of the body – subsequently oxidized or re-esterified in non-adipose tissues [22], [23], for example in the liver. Using stable isotope techniques van Hall et al [24] investigated the triglyceride – FFA cycle, during rest, followed by one hour exercise (60% of VO_2max_) and in the recovery period. Their data indicate that splanchnic (i.e. hepatic) FFA re-esterification is an important factor at rest and immediately after exercise accounting for more than 50% of whole body re-esterification with an increased re-esterification immediately after physical exercise [24]. Based on these data it is tempting to speculate that physical exercise may increase IHCL.

The current study aimed at concomitantly studying the effect of physical exercise on two ectopic fat stores (IMCL, IHCL) in healthy subjects over a broad range of exercise capacity and insulin sensitivity. Based on the hypothesis that the possible changes in ectopic fat depots are mainly regulated by i) exercise capacity and workload, ii) fat availability (subcutaneous and visceral fat mass, systemic free fatty acid concentrations (FFA) and iii) insulin sensitivity (hepatic and peripheral), we investigated both ectopic fat depots using ^1^H-MR-spectroscopy before and after a standardized physical exercise, measured separately subcutaneous and visceral fat mass by multi-slice MR-imaging, FFA concentrations during exercise and assessed peripheral and hepatic insulin resistance using a two-step hyperinsulinaemic euglycaemic clamp.

**Figure 3:**
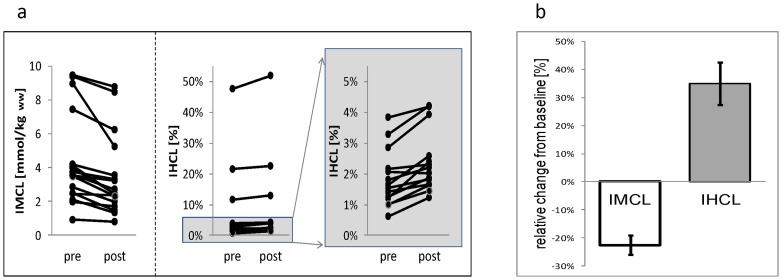
Effect of aerobic exercise on intramyocellular (IMCL) and intrahepatocellular lipids (IHCL). ****Figure 3a**** Individual IMCL and IHCL measurements before (pre) and after (post) a 2h-aerobic exercise at 50% VO_2max_ of each subject. A significant decrease in IMCL (p<0.005) and a significant increase in IHCL (p<0.002) was documented. The diagram on the far right is a blow up of the IHCL data depicted in the middle part to show the changes in IHCL for volunteers with low IHCL levels. **Figure 3b** Changes (mean ± 1 SEM) of IMCL and IHCL (in % from baseline) following a 2h-aerobic exercise at 50% VO_2max._

## Materials and Methods

### (Figure 1)

The protocol for this trial and supporting CONSORT checklist are available as supporting information; see Checklist S1 and Protocol S1. This open, prospective, single-center study was performed at the University Hospital of Bern, Switzerland between September 2007 and May 2009. All investigations were carried out at the Department of Clinical Research (Division of Endocrinology, Diabetes and Clinical Nutrition, and Division of MR- Spectroscopy and Methodology). The study was approved by the local review board (Kantonale Ethikkommission, Bern) and all subjects gave written informed consent. The study was performed according to the declaration of Helsinki, the guidelines of good clinical practice, the Swiss health laws on clinical research (Clinical.Trials.gov: NCT00491582).

**Figure 4:**
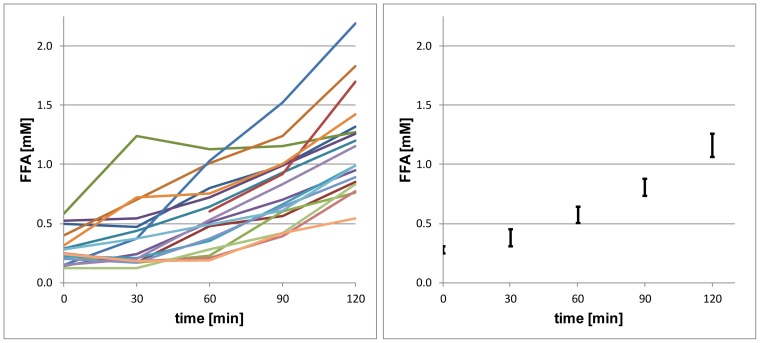
Effect of aerobic exercise on serum free fatty acid concentrations. Individual (left) and overall (mean ± 1 SEM) levels of circulating FFA during the course of the 2 h-aerobic exercise at 50% VO_2max._

### Study participants

Physically active healthy volunteers were included. Criteria for inclusion were a minimum of 30 minutes of exercise 3 times per week and VO_2max_ >50 ml O_2_/kg/min for males and >40 O_2_/kg/min for females and the capacity to perform an aerobic exercise on a treadmill for two hours at 50% VO_2max_. Subjects with contraindications for MR-imaging were excluded.

### Study Protocol (Figure 2)

The subjects attended the endocrine investigation unit for three visits. The maximal time interval between visits was 7 days.

#### Visit 1: Determination of VO_2max_ test on a treadmill

Participants attended the endocrine investigation unit after having fasted for at least 4 hours. All volunteers had restrained from physical activity for 72 hours before the test. Body weight was measured on an electronic balance with subjects wearing light clothes and no shoes. Height was assessed by a stadiometer. End-expiratory waist circumference was measured with a flexible tape placed on a horizontal plane at the level of the iliac crest. Maximal physical capacity was determined during an incremental workload test on a treadmill (CARDIOVIT AT-104 PC Ergo-Spirometrie, Schiller, Baar, Switzerland). Increase of workload was chosen according to the estimated fitness status in order to obtain an exercise time of about 9 – 12 minutes. During the test expired oxygen, carbon dioxide content and minute ventilation were measured continuously (Oxycon alpha, Jaeger, Würzburg, Germany). Furthermore, blood pressure was measured every two minutes and subjective level of exhaustion was assessed with the Borg scale.

After a short break, the subjects began jogging for 60 minutes on the treadmill. This exercise was used to determine the velocity and gradient of the treadmill at which the subject showed an oxygen consumption of 50% of VO_2max_. Furthermore, participants were able to familiarize themselves with the protocol of visit 3.

#### Visit 2: Two-step euglycaemic hyperinsulinaemic clamp

Hepatic and peripheral insulin sensitivity was determined with a two-step hyperinsulinaemic-euglycaemic clamp [25]. Participants arrived at 07:30 after an overnight fast. They had restrained from physical activity for the previous 72 hours and avoided alcohol consumption for 48 hours before the test. On arrival, subjects were asked to void. After that, they rested quietly in a bed in a semisupine position. A cannula was retrogradely inserted into the vein of the left wrist for blood sampling. A second cannula was inserted into an antecubital vein of the other arm for glucose, insulin, and tracer infusions. Blood was collected at baseline for biochemical evaluation. Whole-body glucose turnover was assessed in the basal condition and after a 2-h 6,6-[2H2]glucose infusion (bolus: 3 mg/kg; continuous: 15 μg/kg/min). Afterwards, a 2-step hyperinsulinaemic euglycaemic clamp was performed (Insulin infusion: 0.2 and 1 mU/kg/min, 90 min each) [25]. Stable glycaemia of 5.0 mmol/L was achieved by variable infusion of 20% dextrose according to the actual plasma glucose value which was obtained every five minutes with a bedside glucose meter (YSI2300; Yellow Springs Instruments, Yellow Springs, OH, USA). In order to arterialize venous blood, the sampling hand was heated to 50 – 55 °C in a heated hand box (Dr. Dwight Matthew, University of Vermont). The clamp was performed in combination with measures of hepatic glucose output during the last 30 minutes of each step of the clamp.

#### Visit 3: measurement of IMCL and IHCL before and after a 2 hours physical exercise on the treadmill at 50% of VO_2max_. Determination of subcutaneous and visceral fat mass

During 1.5 days previous to visit 3, subjects followed a fat rich diet consisting of the weight maintaining food intake with a supplementary fat intake of 0.75 g fat/kg BW, administered as 3 additional snacks per day. This protocol had previously been shown to efficiently replete IMCL stores [11]. Upon arrival, they received a standardized light meal. Afterwards IMCL and IHCL were determined by ^1^H-MR-spectroscopy immediately before and after a two hours of walking on the treadmill at a workload of 50% of their VO_2max_. Importantly, transfer times to the MR facility for MR-spectroscopy measurements before and after the physical exercise were 10 minutes at maximum. IHCL were determined first, while IMCL were determined ∼30 minutes later. The decrease in IMCL is defined as ΔIMCL, the increase in IHCL as ΔIHCL. The same day, subcutaneous and visceral fat mass was determined using MR-imaging as described previously [12].

### Biochemical analysis

Blood glucose levels were measured by a glucose-oxidase method using YSI2300 (Yellow Springs Instruments, Yellow Springs, OH, USA). Insulin concentrations were measured with electro-chemiluminescence immunoassays (Roche Modular-E170; Roche Diagnostics, Rotkreuz, Switzerland). FFA concentrations were determined using a commercially available kit (Wako Pure Chemical, VWR International, Dietikon, Switzerland).

### Assessment of hepatic and whole body insulin sensitivity and tracer calculations

Isotopes were produced by Cambridge Isotope Laboratories, Innerberg, Switzerland. Sterile pathogen-free solutions were made by the University Hospital Pharmacy, CHUV, Lausanne, Switzerland. Isotopic enrichments of D-[6-6-2H2] glucose were determined by gas-chromatography mass-spectrometry (GC 5890/MS 5971; Hewlett-Packard, Palo Alto, CA, USA). At rest glucose rate of appearance (Ra) and rate of disappearance (Rd) were calculated from D-[6-6-2H2] glucose enrichments using Steele's equations [26]. Assuming steady state conditions (plasma glucose variation <5%, variation of dextrose 10% infusion <5%) the same equation was used during the last 30 minutes of the first clamp step in order to calculate endogenous glucose production from the infusion rate and the total Ra. As a measure of hepatic insulin sensitivity we report the percentage of suppression of endogenous glucose production in the steady state of the first clamp step compared with the baseline value.

Whole body insulin sensitivity is expressed as glucose infusion per kg body weight during the last 30 minutes of the second clamp step (M-Value).

### Measurement of IMCL and IHCL

The MR examinations were performed on a 3 Tesla system (TIM TRIO, SIEMENS Erlangen, Germany). For the determination of IMCL in m.tibialis anterior, an extremity coil and a PRESS sequence (TR 3s, TE 30ms, 11×12×18 mm3, 128 scans) were used, while for determination of IHCL in segment 6 of the liver a body receive array coil with a STEAM sequence (TR 5s, TE 20 ms, 20×30×30 mm^3^, average of 10 separate non-water-suppressed acquisitions during flat breathing) was applied. Repositioning of the volunteer and placement of the coil for the second measurement after the exercise were monitored on localizer images. For the skeletal muscle, the bottom of the kneecap (patella) and for the liver ,the lower part of the throat (fossa jugularis) was used as a fixation point for the repositioning. For muscle, care was taken that no sign of fatty infiltration was visible inside the sensitive volume, which could lead to contamination by the large extramyocellular lipid signals. [4]. Spectra were analyzed in jMRUI [27]. The 10 acquisitions for IHCL were separately fitted with a fixed pattern of 5 lipid resonances for the region 0.9 to 2.8 ppm. 10 separate acquisitions (1×8 averages, 8×1 average, and 1×32 averages) were used to detect and balance problems of low signal-to-noise (with 1 average) and dephasing (with multiple averages). IHCL results are expressed in signal percent, calculated as % area of the lipid resonances divided by the total signal area (water and lipid resonances in the same, non-water suppressed spectrum). No corrections for relaxation effects were made. IMCL concentrations (in millimoles per kilogram wet weight) were evaluated based on the fully relaxed, unsuppressed water signal as internal concentration standard as previously described [4].

### Subcutaneous and visceral fat mass assessment

MR-imaging was performed on a 3T MR system (TIM TRIO; SIEMENS, Erlangen, Germany) with the body coil as combined transmit/receive coil, as previously described [12]. Briefly, in order to determine visceral and subcutaneous fat mass, images were taken in axial direction with a T1-weighted fast spin echo technique (TR = 452 ms, TE = 38 ms, echo train length = 7, slice thickness of 10 mm, five slices per sequence, spacing between slices 20 mm, FOV 50 cm, image resolution 2 mm per pixel) from fingers to toes leading to 100–130 axial images per subject. Image analysis for volume determination was done by using a home-built program (MATLAB R2007a, The MathWorks, Natick, MA, USA) that is based on an extended point counting method and three sequential steps for the determination of visceral and subcutaneous fat mass [12]: i) the region of visceral fat is separated from subcutaneous fat by a simple contour line, ii) the points for the point counting method are set or deleted by the program based on a threshold value, and iii) visual inspection of the points lets the operator correct for intensity variations resulting from radio frequency inhomogeneity.

### Statistical analysis

Statistical analysis was performed on SPSS 17.0 (SPSS, Inc., Chicago, IL, USA). Results are expressed as mean ± SD. The Kolmogorov-Smirnov test was used to determine the Gaussian distribution of the parameters. Paired t-tests were applied to analyze pre and post-exercise IMCL and IHCL, respectively. Pearson correlations were calculated and univariate regression analysis was performed to examine the influence of fat availability (i.e. subcutaneous and visceral fat mass and systemic FFA), exercise parameters as well as hepatic and peripheral insulin sensitivity on ΔIHCL and ΔIMCL. A p-value <0.05 was considered significant.

## Results

### Clinical and biochemical findings (Table 1)

Data of all subjects were included in the analysis. The clinical parameters, the results of the assessment of fat availability (subcutaneous and visceral fat mass fat mass, systemic FFA), the exercise parameters, the evaluation of hepatic and peripheral insulin sensitivity and the measures of IMCL and IHCL before and after the 2-h aerobic exercise are summarized in Table 1.

**Table 1:** Clinical parameters, body composition and fat availability, exercise parameters, insulin sensitivity and MR-spectroscopy data in endurance trained athletes.

**Clinical Parameters**	
Gender	6 F 12 M
Age (years)	37.6±3.2
BMI (kg/m^2^)	22.5±0.6
Waist	80.4±2.2
**Fat availability**	
Subcutaneous fat mass (kg)	11.7±0.9
Visceral fat mass (kg)	1.7±0.2
Basal FFA (fasting; mmol/L)	0.280±0.032
Peak FFA (during exercise; mmol/L)	1.162±0.099
**Exercise parameters**	
VO_2max_ (ml/kg/min)	53.4±2.9
Workload during 2 h exercise (W)	159.3±10.6
**Insulin sensitivity**	
Suppression EGP from baseline (%; low insulin dose)	64.6±9.07
M-Value (high insulin-dose) (mg/kg/min)	9.7±0.7
**MRS**	
IMCL pre (mmol/kg wet weight)	4.4±0.7
IMCL post (mmol/kg wet weight)	3.4±0.6^β^
ΔIMCL (% from baseline)	−22.6±3.3
IHCL pre (%)	3.6±1.2
IHCL post (%)	4.0±1.3^α^
ΔIHCL (% from baseline)	+34.9±7.6

Values are indicated in mean ± SEM. BMI  =  body mass index; FFA  =  free fatty acids; suppression of EGP  =  suppression of endogenous glucose production  =  measure of hepatic insulin resistance; M-value  =  glucose infusion at high insulin dose  =  measure of peripheral (whole body) insulin resistance; IMCL  =  intramyocellular lipids; IHCL  =  intrahepatocellular lipids expressed as % of hepatic water signal; IMCL pre/IHCL pre  =  before exercise; IMCL post/IHCL post  =  post exercise; ΔIMCL/ΔIHCL  = 2h-aerobic exercise induced changes of IMCL/IHCL; α =  p-value <0.002; β  =  p value <0.001.

### The effect of aerobic exercise on IMCL and IHCL (Table 1, Figure 3a, 3b, 4)

IMCL data of a single patient could not be included in the analysis due to poor quality. A 2h-aerobic exercise at 50%–60% of VO_2max_ resulted in a significant decrease in IMCL (absolute values: −0.96±0.85 mmol/kg wet weight, mean ± SD,; relative values from baseline: −22.6±13.7%, p<0.001) and a significant increase in IHCL (absolute values in signal %): +0.6±0.4%; relative values from baseline: +34.9±32.4%, p<0.002).

### Correlations

Neither ΔIMCL, nor ΔIHCL were significantly correlated with exercise capacity (VO_2max_ and workload), fat availability (subcutaneous and visceral fat mass, systemic FFA concentrations) or insulin sensitivity (hepatic and peripheral).

## Discussion

The main findings of this study can be summarized as follows: a two-hour physical aerobic exercise at 50% of VO_2max_ results in a significant increase in IHCL and decrease in IMCL in the exercising skeletal muscle of healthy subjects. The localization of lipid droplets close to the mitochondria in skeletal muscle suggests that IMCLs are used as a fuel for muscular activity [4]. It is, therefore, neither surprising nor a novel finding that physical activity – as performed in this study – resulted in a significant decrease in IMCL stores. Quantitatively, the current IMCL repletion and depletion following standardized fat diet and physical exercise, respectively, is consistent with previous findings from our group and others [6],[8],[25],[28].

In contrast to IMCL, IHCL significantly increase in healthy human subjects after a 2h-aerobic physical exercise, suggesting that IHCL, like IMCL, are flexible fuel stores. To our knowledge this is a novel finding in humans. It is consistent with studies in rodents (mice and rats) showing that an acute single bout of physical exercise results in an increase in hepatic lipids [29]–[31]. In humans, only indirect evidence – based on stable isotope studies – suggests that splanchnic (i.e hepatic) FFA re-esterfication increases immediately after physical exercise, further corroborating the current results [24]. Mainly long- or intermediate-term interventions over several days or weeks have been performed in humans. They suggest that, like in rodents [28], [32], a high fat diet induces hepatic steatosis [33] whereas an isocaloric low fat diet decreases liver fat content [33], [34]. On the other hand, physical activity without caloric restriction [21] or moderate weight loss after increased physical activity with caloric restriction reduced IHCL [2], [19], [20]. Interestingly, short-term (48h) starvation, which results in a similar increase in systemic FFA concentrations as shown in the present study, also leads to an increase in IHCL further substantiating the flexibility of IHCL [35].

The underlying mechanisms of the regulation of IMCL during exercise are not established. Possible influencing factors include exercise capacity, fat availability and insulin sensitivity. However, exercise parameters (VO_2max_ and workload during exercise), fat availability or markers of insulin sensitivity were not related to ΔIMCL. A possible explanation for these mainly negative findings includes the relatively small number of volunteers. In addition, it may be conceivable that the range of fat mass, exercise capacity and insulin sensitivities was too small in this lean cohort of physically active healthy subjects in order to obtain significant correlations. Alternatively, it may be possible that aerobic exercise is such a strong stimulus that prevails over any other influencing factors. This hypothesis is supported by the fact that a decrease in ΔIMCL could be observed in all subjects independent of age, gender and exercise capacity, as already observed previously [8], [11].

The mechanisms that explain the increase in IHCL during exercise are not established. Cross-sectional studies have repeatedly shown that IHCL assessment without exercise is related to different parameters of insulin sensitivity and visceral fat mass [2], [16]. The current study confirms this fact by establishing a positive correlation between the IHCL levels before exercise and visceral fat mass (data not shown). However, it is intriguing that ΔIHCL was not found to be significantly related to parameters of exercise capacity, fat availability and insulin sensitivity. It can, therefore, only be speculated about possible mechanisms. Most likely the exercise-induced increase in lipolysis far outweighs the cellular transport capacity to take up FFA resulting in a rise in plasma FFA concentrations as shown in this and previous studies [16], [23], [36]. FFA are taken up by the liver and esterified into triglycerides and stored leading to the observed increase in IHCL. This hypothesis is supported by a study in humans using stable isotope techniques and liver biopsies showing that nearly 60% of hepatic triglycerides arose from FFA [37]. It is, therefore, conceivable that by assessing FFA turnover instead of FFA concentrations and fat mass – as in the current study – it would have been possible to establish a significant correlation between ΔIHCL and FFA availability. The exercise-induced increase in IHCL without any relation to hepatic insulin sensitivity may also indicate that the relation between fatty liver and insulin résistance is more complex than suggested by epidemiological studies [16]. Namely, recent studies in animals and humans suggest that the cause-effect relationship between hepatosteatosis and insulin resistance does not exist in all situations [38].

The physiological destiny of the acute surplus of IHCL following exercise still has to be established. However, based on the available evidence [16], [23], [36], [39] it is likely that it is a transitory phenomenon.

The strength of this study is the fact that dietary intake and physical activity were standardized and the latter supervised. In addition, IMCL and IHCL before and after physical exercise were concomitantly assessed, thereby excluding other confounding factors. Finally, factors that may influence the flexibility of ectopic lipids (subcutaneous and visceral fat mass, FFA, insulin sensitivity and exercise capacity) were directly assessed. There are also limitations of this study: 1) physical exercise may change the water content of organs, in particular of the liver thereby changing the reference ^1^H-MR-spectrum. However, even taking into account possible changes of the water signal following exercise the documented changes in IHCL following exercise far offset the potential exercise induced changes of the water signal. 2) The results of the current study are based on a standardized workload (50% of VO_2max_) that may be lower than the intensity at which some of these subjects usually perform. It is, therefore, difficult to extrapolate these data to a higher or lower workload.

In conclusion, the concomitant assessment of IMCL and IHCL suggest that these ectopic lipids are flexible fuel stores that are acutely influenced by physical exercise, albeit in different directions. Whether this phenomenon can also be observed in situations of metabolic or hormonal impairment has to be established.

## Supporting Information

Protocol S1 Trial Protocol
(DOC)

Checklist S1 CONSORT Checklist
(DOCX)
