# Supplementary material for: The Effect of Aerobic Exercise on Intrahepatocellular and Intramyocellular Lipids in Healthy Subjects
Source: PLoS One. 2013 Aug 14;8(8):e70865. doi: 10.1371/journal.pone.0070865 (PMC3743875; doi:10.1371/journal.pone.0070865)
Supplement: Protocol S1 — Trial Protocol (DOC) [file pone.0070865.s001.doc]

Department for Endocrinology, Diabetology and CLinical Nutrition, University Hospital of Bern, Inselspital, CH-3010 Bern

PD Dr. E. Christ

Oberarzt

Bern, den 12.1.2007

Unterschriften

I will conduct the trial as outlined herein.


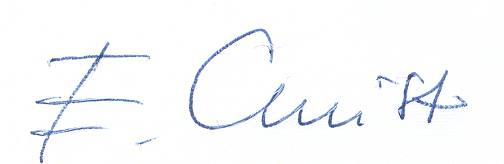


Date PD Dr. med. Emanuel Christ

Principal Investigator

Table of contents

[1. Background and rationale 5](#_Toc156286843)

[1.1 Introduction 5](#_Toc156286844)

[1.2 Diet and fat deposition 6](#_Toc156286845)

[1.3 Exercise and fat deposition 6](#_Toc156286846)

[1.4 GH-deficiency, GHRT, metabolism and nutrition 7](#_Toc156286847)

[1.5 GH-deficiency, GH-replacement therapy (GHRT) and physical activity 7](#_Toc156286848)

[1.6 GH-deficiency, GH-replacement therapy (GHRT) and whole body lipid depots(incl. visceral adiposity) 7](#_Toc156286849)

[1.7 GH-deficiency, GHRT and IMCL (Fig. 1) 7](#_Toc156286850)

[1.8 GH-deficiency, GHRT and IHCL 8](#_Toc156286851)

[2. Hypothesis and endpoints (Fig 1) 9](#_Toc156286852)

[2.1 Primary endpoints of the study are: 11](#_Toc156286854)

[2.2 Secondary endpoints: 11](#_Toc156286855)

[3. Study design 11](#_Toc156286856)

[4. Study population 11](#_Toc156286857)

[4.1 Patients 11](#_Toc156286858)

[4.2 Inclusion criteria 11](#_Toc156286859)

[4.3 Exclusion criteria 11](#_Toc156286860)

[4.4 Control population 12](#_Toc156286861)

[5. Intervention 12](#_Toc156286862)

[6. Schedule, investigation methods and data acquisition 13](#_Toc156286863)

[6. 1 General overview (Figure 3) 13](#_Toc156286864)

[6.1.1 Inclusion, determination of max. exercise capacity and familiarization with study protocol (V1) 13](#_Toc156286865)

[6.1.2 Determination of hepatic and skeletal muscle (peripheral) insulin resistance (V2 and V5) 14](#_Toc156286866)

[6.1.3 Determination of visceral fat mass, repleted and depleted ”ectopic” fat stores, GH-secretion profile (V3 and V6) 16](#_Toc156286867)

[6.1.4 Visits following GHRT (V4-V6) 16](#_Toc156286868)

[6.2 Details on main experiments 16](#_Toc156286869)

[6.2.1 Pre-test standardisation and diaries 16](#_Toc156286870)

[6.2.2 Resting energy expenditure and body impedance analysis (V2 and V5) 16](#_Toc156286871)

[6.2.3 Exercise test (V1 and V3; V4 and V6) 17](#_Toc156286872)

[6.2.4 Clamping (V2 and V5) 17](#_Toc156286873)

[6.2.5 Tracer procedures (V2 and V5) 17](#_Toc156286874)

[6.2.6 Biochemical analysis 18](#_Toc156286875)

[6.2.7 Mass spectrometry analysis 18](#_Toc156286876)

[6.2.8 MRI-Methodology 18](#_Toc156286877)

[6.2.9 Calculations 19](#_Toc156286878)

[6.3 Risk assessment 19](#_Toc156286879)

[6.4 Data management 19](#_Toc156286880)

[7 Statistical analyses 19](#_Toc156286881)

[7.1 Power calculation 19](#_Toc156286882)

[7.2 Calculations of the expected effect 19](#_Toc156286883)

[8. Drug involved in the study 20](#_Toc156286884)

[9. Time table 20](#_Toc156286885)

[10. Ethical and legal aspects, insurance 20](#_Toc156286886)

[11. Responsibilities 20](#_Toc156286887)

[12. Appendix 21](#_Toc156286888)

[12.1 Abbreviations 21](#_Toc156286889)

[13. References: 22](#_Toc156286890)

The effect of growth hormone (GH) on intramyocellular (IMCL), intrahepatocellular lipids (IHCL) and visceral fat mass in relation to insulin resistance

# Background and rationale

## 1.1 Introduction

Insulin resistance is central to obesity, type 2 diabetes, dyslipidaemia and cardiovascular disease. Epidemiological studies indicate that visceral adiposity is a better predictor for insulin sensitivity as assessed by the euglycaemic hyperinsulinaemic clamp than body mass index (BMI) in different ethnic groups (1, 2) suggesting that location of fat deposition - not only the amount of fat - is critical for insulin resistance. Besides intraabdominal fat deposition and increase in lipid content in non adipose tissue (“ectopic”) such as in myocytes (intramyocellular lipids; IMCL) and hepatocytes (intrahepatocellular lipids; IHCL) has been implicated in impaired insulin action (3-7).

IMCL are important fuel stores in skeletal muscle (8, 9). The association between IMCL and insulin resistance has not been fully elucidated. Theoretically, IMCL depend on (i) the amount of free fatty acids (FFA) which is taken up into the skeletal muscle (ii) the capacity of the skeletal muscle to synthesise or, (iii) to degrade IMCL and (iiii) the ability to oxidise FFA. Recent evidence suggest that mainly a reduced capacity to oxidise IMCL contributes to impaired insulin signalling and, therefore, to peripheral insulin resistance (10). The decreased oxidative capacity, in turn, may be related to a decreased number of mitochondria, which may explain the increase in insulin resistance with age (4), or to a dysregulation of IMCL metabolism, which may be caused by an inherited defect in mitochondrial oxidative phosphorylation as shown in healthy young offsprings of parents with type 2 diabetes (3),(11, 12).

An increased hepatic fat content is a common feature in insulin resistance which can ultimately lead to non-alcoholic steatohepatitis (NASH) and cirrhosis (13). However, the underlying pathophysiology is not completely understood. Possible mechanisms include (i) an increase in FFA flux from endogenous stores, especially adipose tissue (subcutaneous and visceral) and their deposition in the liver (14), (ii) an increase in nutritional fat intake (15), (iii) de novo hepatic lipogenesis and (iiii) a reduced hepatic fatty acid oxidation (16). Importantly, recent evidence suggests that the hepatic fat content (IHCL) may be an important determinant of hepatic insulin resistance (i.e. suppression of gluconeogenesis) (17, 18) and can be influenced by weight loss (19) and insulin sensitising agents (6).

Quantitative assessment of IMCL and IHCL using chemical analysis of biopsies was fraught with a large variability (20, 21). Indirect measurements of fat metabolism using indirect calorimetry and tracer methodology was often based on assumptions (22). The non-invasive assessment of IMCL and IHCL using ^1^H-Magnetic resonance spectroscopy (^1^H-MRS) allows to reliably determine IHCL and IMCL quantitatively (20, 21, 23-26). In addition, whole fat distribution including visceral fat tissue distribution can been assessed using MRI-scan (18))

Diet and physical activity impact on insulin action. Insulin, in turn, can be considered as a key hormone regulating lipid metabolism: It promotes hydrolysis of dietary chylomicrones by stimulating lipoprotein lipase activity (27) and inhibiting hormone sensitive lipase in adipose tissue thereby decreasing FFA availability in the circulation (27). Insulin-antagonistic hormones such as GH modulate these actions directly and indirectly and may, therefore, also regulate fat tissue distribution, IMCL and IHCL (28).

Adult patients with GH-deficiency present with features of insulin resistance (29) and hypopituitarism is associated with increased cardiovascular risk (30). Adult GH-deficiency, usually a consequence of a pituitary tumor and its treatment, is a unique clinical condition that allows to study metabolic effects of GH in adult patients.

By administrating GH to GH-deficient patients the impact of GH on fat tissue distribution fat mass, IHCL and IMCL as well as peripheral and hepatic insulin resistance can be assessed. The current knowledge of diet and exercise on fat deposition as well as the impact of GH and insulin on fat mass, IMCL and IHCL is summarized below.

## 1.2 Diet and fat deposition

There is a rapidly growing epidemic of overweight people in the Western world in parallel with insulin resistance and type 2 diabetes (31). Besides physical inactivity high calorie intake contributes to this epidemic (32). On the other hand it is well established that hypocaloric diet results in a reduction of subcutaneous and visceral fast mass (33, 34) in parallel with an improvement of insulin sensitivity (33).

IMCL can be considered as plastic and dynamic fuel reserve within the skeletal muscle that responds to different physiological demands. For example, IMCL stores in physically active subjects are enlarged, rapidly repleted following high fat diet and positively correlated with peripheral insulin sensitivity (35-39). In contrast, sedentary patients with features of insulin resistance (i.e. obesity, type 2 diabetes) exhibit an IMCL content which is increased (like in athletes), but negatively correlated with peripheral insulin sensitivity (40-45). These findings further substantiate the hypothesis that not the amount of IMCL impact on insulin sensitivity but rather the capacity to utilize (i.e. oxidise) IMCL is critical in mediating insulin resistance (16) (46), (47). Importantly, a similar intake of total energy/day, but with a low fat content (15% of energy intake) does not result in an increase in IMCL whereas a high fat diet (i.e. 55% of energy intake) leads to a replenishment of IMCL in healthy volunteers (35) indicating that quality of the diet affects IMCL. In addition, starving for 72 hours (48) resulted in an increase in IMCL in healthy lean volunteers further corroborating the hypothesis that IMCL are rapidly disposable fuel stores which adapt to different physiological situations.

In middle-aged healthy men IHCL has been shown to be tightly correlated with hepatic insulin sensitivity independent of visceral adiposity (49). In women with previous gestational diabetes, recent data indicate that the amount of IHCL is related to total and saturated fat intake suggesting that not only endogenous fat availability but also exogenous fat intake may impact on IHCL (15). These findings are further substantiated by the observation that a hypocaloric diet results in a reduction in subcutaneous and visceral fat mass but also in a substantial decrease in IHCL (19).

## 1.3 Exercise and fat deposition

Physical activity has an important role in the therapy of overweight patients and is a key factor for the prevention of type 2 diabetes (50, 51). If exercise (aerobic or anaerobic) leads to a reduction in weight, the associated fat loss is usually generalised and not confined to visceral adipose tissue (52). Nevertheless, the improved metabolic parameters (i.e. insulin resistance) seen in exercising obese subjects, independent of weight loss, suggest other beneficial actions (52). Possible mechanisms include an effect of exercise on IMCL (47) or IHCL without affecting total weight. Alternatively, modulating hormones, such as GH are known to be secreted during exercise (53) and may, by their metabolic action, impact on lipid stores (54).

IMCL stores are rapidly depleted during aerobic exercise in physically active subjects (35) and moderately active healthy subjects (55). In contrast, in sedentary subjects and patients with characteristic features of insulin resistance the depletion of IMCL with exercise is much less pronounced (56), probably by a decreased capacity to degrade and/or oxidise IMCL. In addition, regular exercise resulted in unchanged (56) or even increased (57) IMCL content. This further substantiates the hypothesis that IMCL are skeletal muscle fuel stores that adapt to different physiological needs.

The impact of physical activity on IHCL in relation to insulin resistance has not been investigated yet.

## 1.4 GH-deficiency, GHRT, metabolism and nutrition

GH has potent metabolic actions. On the other hand, nutrition impacts on GH secretion. GH-deficient patients are usually not diabetic but show clinical and biochemical features of insulin resistance (58, 59). In addition, protein synthesis is impaired (60) and lipolysis is reduced (61). GHRT reverses these abnormalities but further increases insulin resistance, at least after short–term treatment (29).

Conversely, GH secretion is augmented in conditions with low insulin levels (i.e. fasting, anorexia nervosa) whereas in obesity, hyperinsulinaemia is present with an increase in FFA and glucose resulting in a suppression of GH secretion. It is hypothesised that in condition of low energy intake (i.e. fasting) GH shifts energy consumption to lipid oxidation in order to preserve protein mass (62).

## 1.5 GH-deficiency, GH-replacement therapy (GHRT) and physical activity

GH-deficient patient usually present with a reduced exercise capacity and are, therefore, less physically active (63). The reduced lean body mass (29), the reduced performance of the cardiovascular system (64), the reduced oxygen transport capacity (64) and decreased intramyocellular fuel stores (65) may explain these findings.

GHRT has consistently been shown to improve exercise performance (63) mainly by increasing lean body mass (29), improving the performance of the cardiovascular system and oxygen transport capacity (64). Whether the trend for an increase in IMCL following GHRT contribute to the improvement in exercise performance remains to be established (65).

Importantly, exercise is a potent stimulus of pituitary GH secretion in healthy subjects over a broad range of age (53). In addition, GH secretion during exercise is positively correlated with exercise capacity suggesting that GH secretion can be considered as surrogate marker of physical fitness (53).

## GH-deficiency, GH-replacement therapy (GHRT) and whole body lipid depots(incl. visceral adiposity)

Numerous studies have shown that GH-deficient adults have an increased visceral fat mass and, in keeping with this finding, tend to be insulin resistant (29). Interestingly, GH-excess (i.e. patients with GH-secreting pituitary adenoma) is also associated with insulin resistance (66). It is thought that the metabolic action or/and a direct effect of GH is the leading mechanism to induce insulin resistance in acromegalic patients (67) while the increase in visceral fat mass is the main reason for the insulin resistant condition of GH-deficient patients (29). The exact mechanisms, however, are not sufficiently elucidated yet.

Using different techniques (CT scan, MRI) GH replacement therapy (GHRT) has consistently shown to decrease fat mass, in particular visceral fat mass (29, 68). However, although insulin resistance is already slightly increased in the GH-deficient patients it usually increases further with GHRT after short-term treatment (6 weeks) and returns to baseline values following 12-16 weeks therapy (69).

## 1.7 GH-deficiency, GHRT and IMCL (Fig. 1)

So far, there is no published data available on IMCL in relation to GH-deficiency or GHRT. The preliminary results of the previous SNF grant (No 3200BO-100146) with 13 patients before and after GHRT suggest that IMCL tend to be low after negative energy balance (exercise and low fat diet) and are rapidly repleted following high fat diet and physical inactivity (Fig. 2). The changes in IMCL content between the depleted and repleted condition are significantly increased in GH-deficient patients compared to normal matched controls (Fig. 3). This is in contrast to findings in overweight patients with insulin resistance without pituitary disease (42).

GHRT tended to increase IMCL content following exercise and low fat diet whereas repletion of IMCL was similar before and after GHRT. In parallel, postprandial metabolic measurements (glucose, triglycerides, insulin) suggest an increase in insulin resistance following GHRT in keeping with an earlier observation (70). Theoretically, an increased uptake of FFA and/or IMCL synthesis or a decrease in IMCL-utilisation could explain these results. Taken together these results confirm the actual concept that IMCL does not mediate insulin resistance per se and imply that besides exercise and diet GH may modulate IMCL content.

## 1.8 GH-deficiency, GHRT and IHCL

Indirect evidence using tracer methodology and the two-step hyperinsulinaemic euglycaemic clamp technique indicates that hepatic insulin resistance is impaired in GH-deficient patients (54). IHCL has been shown to be associated with serum triglycerides (TG) in obese women with previous gestational diabetes (15). GH-deficient patients often present with a dyslipidaemia characterised by an increase in TG and LDL-cholesterol (LDL-C) concentrations (29). In addition, turnover data indicate that hepatic VLDL apolipoprotein B100 (VLDL apoB) production is increased in GH-deficiency (71), consistent with findings in insulin resistant patients with type 2 diabetes (72). Since VLDL apoB secretion depends on intrahepatocellular lipid availability (73) these data indicate that IHCL may be increased in GH-deficiency. Finally, an increase in FFA flux from the enhanced visceral fat stores to the liver may contribute to the increase in hepatic lipid availability (68). Taken together, dyslipidaemia, turnover data and enhanced visceral fat stores suggest that IHCL may be increased in GH-deficiency. Reliable data on IHCL, however, are currently still lacking.

Short-term GHRT usually results in an increase in hepatic insulin resistance (54) in parallel with unchanged or slightly elevated TG levels whereas LDL-C usually decreases (29). VLDL apoB turnover data show a further increase in VLDL apoB production in parallel with an increase in VLDL apoB catabolism resulting in an unchanged VLDL apoB pool (74). Whether GHRT affects IHCL remains to be established.

Interestingly, recent observational data indicate a high prevalence of non-alcoholic fatty liver disease ultimately leading to cirrhosis and transplantation among patients with pituitary or hypothalamic disease (75). This underlines the importance of assessing IHCL and hepatic insulin resistance in patients with pituitary disease.

Figure 1

Changes in intramyocellular lipid content (repleted – depleted) in 13 GH-deficient patients before and after GH-replacement therapy and in matched control subjects

# p=0.07

# p=0.07

p<0.02

GHD delta = IMCL repleted - IMCL depleted in GH-deficient patients

# 2. Hypothesis and endpoints (Fig 1)

Visceral fat mass and “ectopic” fat deposition (IMCL, IHCL) interferes with insulin action. Physical activity and nutrition impact on lipid stores. The GH/IGF-I axis is a modulator of lipid stores, thereby regulating insulin sensitivity.

Patients with GH-deficiency have normal beta-cell function but an increase in visceral obesity. Due to the augmented availability of FFA within the portal system IHCL will be increased compared to control subjects. In contrast, due to the lack of the GH-induced lipolytic action on adipose tissue with a concomitant decrease in FFA availability within the peripheral circulation, IMCL will be reduced. Accordingly, hepatic insulin resistance will be mainly impaired. GH replacement therapy will reverse these changes.

Figure 2

Working hypothesis

Growth hormone, possible impact of “ectopic” fat accumulation and insulin resistance

##

Physical activity

Diet

## Body fat stores

- Visceral fat mass
- Intramyocellular lipids (IMCL)
- Intrahepatocellular lipids (IHCL)

Hepatic and peripheral insulin resistance

GH status

Physical activity and quality of diet impact on visceral fat mass, intramyocellular lipids (IMCL) and intrahepatocellular lipids (IHCL). These fat stores are associated with hepatic and peripheral (skeletal muscle, adipose tissue) insulin resistance. Hormones impact on energy homeostasis by affecting exercise performance and/or regulating fuel metabolism, thereby adapting the subject to changing exogenous challenges. GH affects physical activity by improving cardiovascular performance. Metabolic effects of GH are anabolic relating to protein metabolism (increase in lean body mass), catabolic regarding fat metabolism (decrease in fat mass) and inducing insulin resistance in connection with carbohydrate metabolism. In contrast, hypoglycaemia, falling free fatty acids and increased amino acids stimulate pituitary GH release.

GH-deficient patients present with an increase in visceral fat mass and a tendency for reduced IMCL. GH-replacement therapy (GHRT) results in a decrease in visceral fat mass and a trend for an increase in IMCL. The effect of GH-deficiency and GHRT on IHCL is unknown.

## 2.1 Primary endpoints of the study are:

1. To define visceral fat mass and “ectopic” fat deposition in patients with GH-deficiency in response to exercise and diet compared to control subjects and physically active subjects.
2. To investigate the association between “ectopic” fat deposition and measurements of peripheral and hepatic insulin resistance in patients with GH-deficiency compared to control subjects and physically active subjects.
3. To determine the effect of GHRT in GH-deficient patients on “ectopic” fat stores in response to exercise and diet.
4. To examine the association between changes in “ectopic” fat stores following GHRT and changes in systemic measurement of peripheral and hepatic insulin resistance.

## 2.2 Secondary endpoints:

1. to assess the physical activity in GH-deficient patients compared to sedentary control subjects and physically active subjects

2) to measure the effect of GHRT on exercise capacity in GH-deficient patients

#

# 3. Study design

This is a prospective single-centre open case-control study. The study will be performed according to the declaration of Helsinki, the guidelines of good clinical practice (GCP), the Swiss health law (HMG dated 15 December 2000 and 27 November 2001) as well as the ordinance on clinical research (dated 17 October 2001). The study will be performed at the University Hospital of Bern. Investigations are located in the rooms of the Division of Endocrinology, Diabetes and Clinical Nutrition (located in the Children’s Hospital, U1) and in the MRI-Centre

# 4. Study population

## 4.1 Patients

Patients with hypopituitarism of the outpatient clinic of the division of Endocrinology and Diabetes of the University of Berne, Inselspital, will be tested for GH-deficiency according to the recommendations of the growth hormone research society (76). A total of 18 patients with adult GH-deficiency will be recruited.

## 4.2 Inclusion criteria

- Male and female patients with proven GH-deficiency defined as a peak GH of less than 3mU/l during an insulin provocation test with nadir plasma glucose less than 2.2 mmol/l and additionally, stable conventional replacement therapy including corticoids, thyroxin and gonadale hormones as needed.
- Ability to perform an exercise test on a treadmill or a walking band.
- Willingness to participate in the study and to give written informed consent.

##

## 4.3 Exclusion criteria

- Active neoplasia
- Severe cardiovascular disease (unstable coronary heart disease, heart failure NYHA III-IV)
- Type 2 Diabetes mellitus
- Haemophilia or other coagulation disorder
- Inability to exercise
- Contraindications to exposure to a 3-T magnetic field (Pace-Makers, osteosynthetic material)
- Pregnant women
- Women in childbearing age unless on a continuous contraceptive therapy or surgically sterilised.
- Abnormal liver or renal function (Creatinine >130μmol/L, normal reference 45-93μmol/L; ASAT and ALAT > 3 times the upper reference limit).
- Major depression, psychosis and other severe personality disorders
- Excessive alcohol consumption (>60g/d) or drug-abuse
- Refusal to give written consent
- Patients, who are not suitable for the study according to the study physician

## 4.4 Control population

Twenty age, gender and BMI matched sedentary (without regular sports activity) subjects will be recruited.

Twenty physically active healthy volunteers will be studied. Criteria for inclusion are: minimum 30’ of exercise 3 times per week and VO_2 max_ > 50 ml O_2_/kg/min for males and >40 O_2_/kg/min for females.

# 5. Intervention

Therapeutical intervention include GHRT in GH-deficient adults. This indication is accepted by Swissmedic. In addition a dietary intervention (additional fat snacks) and a controlled physical activity (determination of VO_2max_ and cycling at 50% VO_2max_ ) will be performed.

# 6. Schedule, investigation methods and data acquisition

## 6. 1 General overview (Figure 3)

Figure 3 Clinical Protocol

= equilibrated weight maintaining diet

= minimum 7 days between V1 and V2. no physical activity, equilibrated weight maintaining diet

= minimum 7 days between V2 and V3. High fat diet three days before V3**;** No exercise, food diary.

= usual clinical care: 6 months hypopituitary patients: monthly visits (IGF-I and Bioimpedance measurements)

V1 = Visit 1: determination of VO_2max_ (Spiroergometry). Nutritional advice. Food diary, Familiarization with study protocol (i.e. treadmill)

V2 = Visit 2 Two-step hyperinsulinaemic euglycaemic clamp with stable isotopes (d-[U-^13^C]glucose and d-[6-6-^2^H_2_]glucose)

V3 = Visit 3 MRS/MRI (IMCL, IHCL, visceral fat mass), repleted.

1.5 hour physical activity (bicycle) at VO_2max_ 50-55%, GH secretion profile

MRS/MRI (IMCL, IHCL), depleted

V4 identical to V1 without familiarization

V5 identical to V2

V6 identical to V3

V1

V2

V3

V4

V5

V6

Identical investigations will be performed before and after an intervention. GH-deficient patients will be studied at baseline and after 6 months GH replacement therapy. Sedentary matched control subjects and physically active subjects (road or cross-country cyclists, duathletes, and triathletes; VO_2max_ > 50mL/O_2_/kg) will be tested once.

Investigations include a) determination of exercise capacity (VO_2max_), b) measurements of “ectopic” fat deposition (IMCL, IHLC) and visceral fat mass using MRS and MRI, respectively, in repleted (no physical activity, high fat diet) and depleted (after physical activity) condition. GH secretion profile will be obtained during a standardised exercise. c) hyperinsulinaemic euglycaemic clamp in combination with stable isotope technique and indirect calorimetry to measure hepatic and skeletal muscle insulin resistance and fuel oxidation (77).

### 6.1.1 Inclusion, determination of max. exercise capacity and familiarization with study protocol (V1)

The purpose of the study will be explained to the patient and informed consent will be obtained. Information on personal details and sport related history will be collected (especially relevant to inclusion criteria). A physical examination which includes basic anthropometric (height, weight, and waist circumference) measurements will be carried out.

Afterwards the maximal physical capacity (VO_2max_) will be determined. The usual therapy will be administered prior to exercise, where appropriate. Subjects will have fasted for at least 6 hours and restrained from strenuous activity for at least 72 hours. VO_2max_ will be determined during an incremental workload test to exhaustion with a brake bicycle Ergometer (CARDIOVIT AT-104 PC Ergo-Spirometrie, Schiller, Baar, Switzerland). The fitness test will be combined with continuous analysis of expired oxygen, carbon dioxide content and minute ventilation using a breath by breath analysis (Oxycon alpha, Jaeger, Würzburg, Germany) and under continuous ECG control.

The patients and sedentary control subjects will begin at a workload of 20 watts, which will be gradually increased by 10-20 watts every minute until exhaustion. The increase in workload is chosen in order to obtain an exercise time of 10-12 minutes in these sedentary subjects. The physically active subjects will begin at a workload of 80 watts.

Afterwards the patients and the sedentary and physically active subjects controls will have a light standardised breakfast before cycling on a treadmill at 50%-55% of VO_2max_ for 90 minutes (= familiarization with the study protocol).

### 6.1.2 Determination of hepatic and skeletal muscle (peripheral) insulin resistance (V2 and V5)

After a fasting blood sampling (GH, IGF-I, cortisol, sex steroids, thyroid hormones, insulin, FFA, glycerol, lipid profile, glucose), a two step hyperinsulinaemic, euglycaemic (5 mmol/L) clamp will be performed (Human Actrapid, Novo Nordisk, Copenhagen, Denmark; low dose insulin: 0.4 mU/kg/min; high dose insulin: 1mU/kg/min) for total 4 hours (each insulin dose 2h). Glucose will be measured every five minutes. Insulin and FFA levels every 15 minutes. Samples for enrichment (glucose) will be taken during the last 30 minutes of each insulin step. The glucose infusion requirements to maintain euglycaemia are recorded. Plasma glucose concentrations are then maintained with an infusion of 20% glucose derived from potato starch, which is recognized to have low ^13^C enrichment. The glucose infusion is spiked with d-[U-^13^C]glucose and d-[6-6-^2^H_2_]glucose to maintain steady enrichment values (hot infusate technique) to avoid dilution of tracer by the glucose infusion (78). Indirect calorimetry will be carried out to assess oxidation rate at baseline and every 30 minutes.

Instructions for a high fat diet will be given at the end of visit 2. This consists of the usual food intake with a supplementary fat intake of 0.75g fat/kg BW, administered as 3 additional snacks per day for three days. A list of suitable fat enriched snacks will be given to the patient. This protocol has previously been shown to efficiently replete IMCL stores (35, 65). A food diary will be kept. The patient will be asked to restrain from physical activity. This will be monitored by a pedometer (Digi-Walker®) throughout the whole period (aim < 5000 paths/24 hours).

Figure 4. Determination of hepatic and skeletal muscle (peripheral) insulin resistance. Tracer and blood sampling schedule

Plasma Insulin, FFA and glycerol concentrations every 15 minutes

Estimated blood volume/clamp: 175 ml

Background enrichment (3x) samples

(air and plasma glucose)

enrichment samples (4x)

(air and plasma glucose)

indirect calorimetry

Plasma glucose measurements every 5-10 minutes

- Tracer Infusion (during clamp with hot infusion technique)
- d-[U-^13^C]glucose 0.8µmol·kg^-1^·h^-1^, primed with 0.6 µmol NaH^13^CO_3_ and 1.2µmol/kg d-[U-^13^C]glucose
- d-[6-6-^2^H_2_]glucose 0.13 µmol/kg/min, primed with 12 µmol/kg

Baseline period (90 min.)

# Low dose insulin

(0.4mU/kg/min) (120min.)

High dose insulin

(1mU/kg/min) (120 min)

0 60 90 180 210 300 330 min

### 6.1.3 Determination of visceral fat mass, repleted and depleted ”ectopic” fat stores, GH-secretion profile (V3 and V6)

The patients will attend the hospital after an overnight fast. After a fasting blood sampling (GH, IGF-I, cortisol, thyroid hormones, insulin, FFA, glycerol, lipid profile, glucose), measurements of total body composition (bioimpedance) and indirect calorimetry, patients and control subjects will receive a conventional standardised breakfast (carbohydrates 50%, Protein 20%, Fat 30%) according to 30% of the necessary energy intake (based on indirect calorimetry). Usual medication will be administered, where appropriate. “Ectopic” fat stores (IMCL, IHCL and whole body fat distribution incl. visceral fat mass will be measured by MRS and MRI, respectively (repleted condition).

Subsequently, patients and control subjects will exercise at 50 % of VO_2max_ for 1.5 hour. Our data (36) and results of the literature (55) suggest that IMCL are already reduced after 1 hour aerobic exercise in sedentary subjects. During the exercise test samples are taken for GH secretion profile (every 15 minutes). IMCL, IHCL and whole body fat distribution will be re-assessed following exercise, (“ectopic” fat stores depleted).

Patients will be instructed in self-administration of GH.

The control subjects will be investigated once.

### 6.1.4 Visits following GHRT (V4-V6)

Usual clinical care will be provided (monthly visit with measurements of body composition (bioimpedance), plasma IGF-I and glucose levels to adjust GH doses in GH-deficient patients. GH dose will be gradually increased in order to obtain IGF-I concentrations in the upper half of the age-adjusted normal range as suggested by the growth hormone research society (76). After six months of GH replacement therapy with a concomitant weight maintaining diet and usual physical activity identical studies will be performed (diet and physical activity matching the first study). V4 corresponds to V1 without familiarization of the study protocol. V5 and V6 matches V2 and V3, respectively.

## 6.2 Details on main experiments

### 6.2.1 Pre-test standardisation and diaries

To control for confounding by external factors relevant to the de- and repletion of lipid stores (such as diet and exercise) detailed diaries will be filled out by patients beginning 1 week in advance of V1 and will be continued until V3. The diaries cover the following items:

- Food ingestion (time, quantity of (at least) carbohydrates, and composition)
- Exercise bouts (Quality, duration, grade, time), pedometer readings

Patients are instructed to eat an isocaloric diet (35-40 kcal/kg body weight; 50-55% carbohydrate, 20% protein, and 20-25% fat). Ingested calories (separated for carbohydrates and lipids) will be calculated from nutrition tables. Strenuous exercise is not allowed 72 h before the test days V1 and V4. Similar activity levels will be monitored by a pedometer (Digi-Walker®) throughout the same period (aim < 5000 paths/24 hours).

### 6.2.2 Resting energy expenditure and body impedance analysis (V2 and V5)

After an overnight fast resting energy expenditure (REE), respiratory exchange ratio (RER), oxygen consumption (VO_2_) and carbon dioxide production (VCO_2_) will be assessed by indirect calorimetry during 20 minutes (DeltatracTM II, AVL Medical Systems AG, Schaffhausen, Switzerland). Rates of substrate oxidation will be calculated using standard equation (79).

Body composition will be estimated by body impedance analysis (BIA 1001/S, RJL systems, Detroit, U.S.A.) after voiding of urine.

### 6.2.3 Exercise test (V1 and V3; V4 and V6)

Each patient will cycle during 90 min at a power which corresponds to 50-55% of VO_2max_ on a cycle ergometer (CARDIOVIT AT-104 PC Ergo-Spirometrie, Schiller, Baar, Switzerland). Patients are asked to pedal with a frequency of 70-90 rpm. Cooling will be performed by fan; the room temperature is kept constant at 20-22°C. There will be no warming up and no encouragement.

- On indirect calorimetry RER, VO2, and VCO2 are measured continuously. Heart Rate is measured continuously with peripheral ECG-leads. At the beginning and at the end of the exercise the patient is asked to empty his bladder. Every 15 minutes RPE is assessed by the Borg scale (by the same investigator).

All procedures will be repeated at the same time of the day and on the same days of the week.

### Clamping (V2 and V5)

The cannula for blood sampling will be placed into a thermo-stabilized box heated at 60°C to achieve partial arterialisation of venous blood. The glucose infusion requirements to maintain euglycaemia (or hyperglycaemia) are recorded. Plasma glucose concentrations are then maintained with an infusion of 20% glucose derived from potato starch, which is recognized to have low ^13^C enrichment. The glucose infusion is with d-[U-^13^C]glucose and d-[6-6-^2^H_2_]glucose to maintain steady enrichment values (hot infusate technique). Glucose will be measured every five minutes (arterialised blood, centrifugated for 3 min at 10.000 rpm, glucose oxidase method in 2300 STAT plus, YSI, Yellow Springs Instruments, Yellow Springs, Ohio, OH).

Regular insulin (Actrapid, Novo Industries, Copenhagen, Denkmark) in isotonic saline is infused in a constant manner with a calibrated volumetric pump (MCM 500, MC Medizintechnik, Alzenau, Germany) at two doses (low dose insulin: 0.4 mU/kg/min; high dose insulin: 1mU/kg/min) The targets for blood glucose levels are set to 5 mmol/L

### 6.2.5 Tracer procedures (V2 and V5)

The following tracers will be administered via calibrated volumetric pumps (MCM 500, MC Medizintechnik, Alzenau, Germany; Harvard 22, Harvard Apparatus, South Natick, MA, USA; Perfusor secura FT, Braun, Sempach, Switzerland) beginning at 8.00 am together with clamping:

- d-[U-^13^C]glucose at a rate of 0.8µmol·kg^-1^·h^-1^, primed with 0.6 µmol NaH^13^CO_3_ and 1.2µmol/kg d-[U-^13^C]glucose (80) to target an enrichment of 0.05%;
- d-[6-6-^2^H_2_]glucose at a rate of 0.13 µmol/kg/min, primed with 12 µmol/kg to target an enrichment of 1.25%;

Baseline blood and breath samples will be taken prior to infusion for measurement of background enrichment. After a 60-min equilibration period to reach steady-state tracer enrichment, the basal steady state will be sampled prior to exercise (-30 to 0 min). The hot infusate technique is used to avoid dilution of tracer by the glucose infusion (81) as described under 6.2.6. All tracers are kindly provided by Prof. L. Tappy, CHUV, Lausanne. Isotopes are manufactured by Cambridge Isotope Laboratories, Innerberg, Switzerland.

### Biochemical analysis

Glucose concentrations in plasma will be determined by glucose analyser (Yellow Spring Instruments, Yellow Springs, OH, U.S.A). Plasma FFA and fasting lipid profile will be determined using commercially available kits (FFA: Wako Chemicals, Germany, TG, total cholesterol: enzymatic method Boehringer, Mannheim, Mannheim, Germany; HDL-cholesterol: Wako Chemicals, Germany). These analysis will be performed in the laboratory of Prof. L. Tappy, CHUV, Lausanne. Plasma insulin, GH and IGF-I concentrations will be measured by chemiluminescent methods (Immulite^®^). Sex steroids will be determined by solid phase RIA and thyroid hormones will be determined by competitive immunoassay using direct chemiluminescent technology (Chiron Diagnostics, East Walpole, MA, U.S.A). All hormone analysis will be performed in the central chemical laboratory of the Teaching Hospital of Berne (Inselspital) or in the laboratory of Prof. PE Mullis, Children’s University Hospital of Berne (Inselspital). Serum glucose and lactate is measured by an immobilised glucose-oxidase method (YSI 2300, Yellow Springs Instruments, Yellow Springs, OH, U.S.A.).

### 6.2.7 Mass spectrometry analysis

All isotopic enrichments of glucose and CO_2_ will be determined by gas-chromatography mass-spectrometry (GC 5890/MS 5971, Hewlett-Packard, Palo Alto, CA, U.S.A) in collaboration with the laboratory of Prof. L. Tappy, CHUV, Lausanne.

### MRI-Methodology

Magnetic resonance examinations will take place at the MR centre of the Inselspital and University of Bern, performed by the MR section of the Department of Clinical Research (AMSM). MR exams include hepatic (IHCL) and intramyocellular (IMCL) lipid concentrations measured by ^1^H magnetic resonance spectroscopy (^1^H-MRS) and liver volume and whole body fat distribution will be determined by means of magnetic resonance imaging (MRI).

^1^H-MRS before (repleted) and after (depleted) exercise (V2 and V5) will be performed on a clinical 3 T whole body scanner (Magnetom, Trio, Siemens, Germany) equipped with a ^13^C/^1^H double tuned flexible coil (1H: Helmholtz design, Medical Advance). The right leg of a patient will be placed in a specially designed cast to guarantee a reproducible position and shape of vastus intermedius muscle. Positioning of the muscle, fixation of the surface coil, and placement of the ^1^H-voxel will be monitored by two series of localizer images. The same images will be used to calculate total muscle volume. MRS data will be acquired using an optimised press sequence (repetition time = 3 s, echo time = 20 ms, 128 acquisitions, water presaturation, outer volume suppression) and quantified using the unsuppressed fully relaxed MR signal as internal standard. Absolute IMCL levels in mmoles/kg muscle wet weight will be calculated as reported earlier (82). A reproducibility study revealed a typical CV of 6% for repeated measurements (83).

In order to detect potential changes of the liver size and subsequent variations of the total amount of IHCL, liver volume will be determined in end-expiratory breath hold by established MRI sequences that have already been successfully applied by the AMSM (84). In muscle, liver and whole body studies, Cavalieri principles with point counting have been used (84), (85), (86) to determine organ volumes reliably.

Whole body fat distribution incl. visceral fat mass will be determined using the methodology described by Machann J. et al (2005). Briefly, an axial T1-weighted fast spin echo technique with an echo train length of 7 will be applied. Measurements parameters: TE/TR 12 msce/490 msec, slice thickness 10 mm, 5 slices per sequence, 10 mm gap between the slices. The field of view will be 450 mm to 530 depending on the size of the volunteer. A 256 x 178 matrix will be recorded in a measuring time of 12 seconds, allowing breath-hold examinations in abdominal regions. Table shift will be set at 10 cm. Volunteers are in prone position with the arms extended and data are collected from fingers to toes. In total, 100 to 130 images will be obtained depending on the size of the volunteer.

### Calculations

Glucose appearance and disappearance rate will be calculated from d-[6-6-^2^H_2_]glucose, d-[U-^13^C]glucose (internal control) isotopic enrichments, respectively, using Steele’s equations (87) at baseline, after low and high dose of insulin. Glucose oxidation will be calculated from labelled ^13^CO_2_ and labelled d-[U-^13^C]glucose. The volumes of distribution of glucose is assumed to be 100 ml/kg. The bicarbonate pool is assumed to be 14.2 mmol/kg body weight.

## Risk assessment

In this population exercise at the proposed level can be considered safe. The tracer technology using stable isotopes is considered safe with the proposed concentrations. MRI is a non-invasive technology.

## Data management

All subjects’ personal data will be treated confidentially. Only anonymised results will be published or passed on to third parties. Ethics Committee members or Swiss Regulatory Authorities may require to have access to the medical notes of the patients. In this case, these persons will respect the confidentiality of the private data of the study subjects

# Statistical analyses

## Power calculation

There is no data available about IHCL and GH-deficiency. In our previous study (14 GH-deficient patients, 11 control subjects) waist circumference was significantly increased in patients compared to control subjects and significantly decreased (by 2-3 cm) after GHRT suggesting that abdominal fat tissue significantly changes. By including 18 patients we shall have enough power to detect a difference in intraabdominal fat mass. With regard to IHCL the power calculation is,based on IHCL data obtained from patients with type 2 diabetes, which is also an insulin resistant condition (5). . In these patients changes in IHCL were observed after therapy with Rosiglitazone (an insulin sensitizer). It can be estimated that with the paired study design a similar reduction of IHCL (3.6 percent liver mass; SD 4.41, conservatively calculated from *p*-value) would be detected following GHRT with a power of 90% in a sample size of 18 subjects.

## Calculations of the expected effect

Data will be presented as the mean ± SD. Paired t-test will be performed in patients to assess the effect of therapeutical intervention (GHRT). Unpaired t-test will be performed between GH-deficient patients before and after GHRT and control subjects (Atheltes and sedentary controls). In particular ectopic fat depots (i.e. IHCL; IMCL, delta IMCL and visceral fat mass) will be compared before and after GHRT. Unpaired t-test will be used to analyse differences in ectopic fat depots between GH-deficient patients, sedentary control subjects and physically active subjects. Parameters, which are not normally distributed will be log transformed prior to analysis. Alternatively non-parametric tests will be used. Correlation analysis between Ectopic fat depots and measurements of insulin resistance (clamp data) will be performed using Spearman’s rank correlation coefficients. P<0.05 will be considered significant. Statistical analyses will be performed on Stata 9.1 (Stata Corporation, College Station, TX, USA), and Microsoft Excel (Microsoft Corporation, USA).

# 8. Drug involved in the study

GHRT will be performed with Genotropin^®^ (Pfizer, Oerlikon, Switzerland) using a special pen device. The indication for GHRT ind GH-deficient patients is accepted by Swissmedic and the health insurance companies (no off label use). Genotropin^®^ will be provided by Pfizer and will be stored in the refrigerator (permanent temperature control) of the department of Endocrinology , Diabetology and Clinical Nutrition, Inselspital Bern. Instruction to utilize and store Genotropin^®^ will be provided to the patients as in usual clinical care.

# 9. Time table

Recruitment (including controls) March 2007 – September 2009

Number of GH-deficient patients: 18

Number of controls: 20 sedentary volunteers, 20 physically active healthy subjects

Number of Visits GH-deficient patients: 18x6 = 108

Number of visits sedentary controls: 20x3 = 60

Number of visits physically active subjects: 20x3 = 60

# 10. Ethical and legal aspects, insurance

The study protocol will be presented to “Kantonale Ethikkommission des Kantons Bern”. The study is in accordance with the respective ethical standards for human investigation and with the Helsinki Declaration of 1975, as revised in 1983.

The usual clinical visits (not study investigations) will be covered by the health insurance companies. All the other investigations are covered by study funding.

The insurance company of the University Hospital of Bern, Inselspital, is responsible for all problems with possible side effects of the study.

# 11. Responsibilities

Study-physicians of the Division of Endocrinology and Diabetology, Inselspital, Bern.

Main investigator: PD Dr. med. Emanuel Christ

Research fellow: Dr. med. Andrea Egger

# Appendix

## 12.1 Abbreviations

| V  GH  IGF-1 | Visit  Growth hormone  Insulin-like growth factor-1 |
| --- | --- |
| GHD | Growth hormone deficiency |
| GHRT  IMCL | Growth hormone replacement therapy  Intramyocellular lipids |
| IHCL | Intrahepatocellular lipids |
| VO_2max_  REE | maximal Oxygen uptake  resting energy expenditure |
| RER  V O_2_  VCO_2_  ^1^H-MRS  MRI  FFA  LDL-C  TG | respiratory exchange ratio  oxygen consumption  carbon dioxide production  ^1^H-Magnetic resonance spectroscopy  Magnetic resonance imaging  Free fatty acids  LDL-cholesterol  Triglycerides |
|  |  |
|  |  |
|  |  |
|  |  |
|  |  |
|  |  |
|  |  |

# References:

1. Carey DG, Jenkins AB, Campbell LV, Freund J, Chisholm DJ 1996 Abdominal fat and insulin resistance in normal and overweight women: Direct measurements reveal a strong relationship in subjects at both low and high risk of NIDDM. Diabetes 45:633-8

2. Fujimoto WY, Bergstrom RW, Leonetti DL, Newell-Morris LL, Shuman WP, Wahl PW 1994 Metabolic and adipose risk factors for NIDDM and coronary disease in third-generation Japanese-American men and women with impaired glucose tolerance. Diabetologia 37:524-32

3. Petersen KF, Dufour S, Befroy D, Garcia R, Shulman GI 2004 Impaired mitochondrial activity in the insulin-resistant offspring of patients with type 2 diabetes. N Engl J Med 350:664-71

4. Petersen KF, Befroy D, Dufour S, Dziura J, Ariyan C, Rothman DL, DiPietro L, Cline GW, Shulman GI 2003 Mitochondrial dysfunction in the elderly: possible role in insulin resistance. Science 300:1140-2

5. Mayerson AB, Hundal RS, Dufour S, Lebon V, Befroy D, Cline GW, Enocksson S, Inzucchi SE, Shulman GI, Petersen KF 2002 The effects of rosiglitazone on insulin sensitivity, lipolysis, and hepatic and skeletal muscle triglyceride content in patients with type 2 diabetes. Diabetes 51:797-802

6. Bajaj M, Suraamornkul S, Pratipanawatr T, Hardies LJ, Pratipanawatr W, Glass L, Cersosimo E, Miyazaki Y, DeFronzo RA 2003 Pioglitazone reduces hepatic fat content and augments splanchnic glucose uptake in patients with type 2 diabetes. Diabetes 52:1364-70

7. Anderwald C, Bernroider E, Krssak M, Stingl H, Brehm A, Bischof MG, Nowotny P, Roden M, Waldhausl W 2002 Effects of insulin treatment in type 2 diabetic patients on intracellular lipid content in liver and skeletal muscle. Diabetes 51:3025-32

8. Boesch C, Slotboom J, Hoppeler H, Kreis R 1997 In vivo determination of intra-myocellular lipids in human muscle by means of localized 1H-MR-spectroscopy. Magn Reson Med 37:484-93

9. Schick F, Eismann B, Jung WI, Bongers H, Bunse M, Lutz O 1993 Comparison of localized proton NMR signals of skeletal muscle and fat tissue in vivo: two lipid compartments in muscle tissue. Magn Reson Med 29:158-67

10. Shulman GI 2000 Cellular mechanisms of insulin resistance. J Clin Invest 106:171-6

11. Jacob S, Machann J, Rett K, Brechtel K, Volk A, Renn W, Maerker E, Matthaei S, Schick F, Claussen CD, Haring HU 1999 Association of increased intramyocellular lipid content with insulin resistance in lean nondiabetic offspring of type 2 diabetic subjects. Diabetes 48:1113-9

12. Perseghin G, Scifo P, De Cobelli F, Pagliato E, Battezzati A, Arcelloni C, Vanzulli A, Testolin G, Pozza G, Del Maschio A, Luzi L 1999 Intramyocellular triglyceride content is a determinant of in vivo insulin resistance in humans: a 1H-13C nuclear magnetic resonance spectroscopy assessment in offspring of type 2 diabetic parents. Diabetes 48:1600-6

13. Silverman JF, Pories WJ, Caro JF 1989 Liver pathology in diabetes mellitus and morbid obesity. Clinical, pathological, and biochemical considerations. Pathol Annu 24 Pt 1:275-302

14. Wahrenberg H, Lonnqvist F, Arner P 1989 Mechanisms underlying regional differences in lipolysis in human adipose tissue. J Clin Invest 84:458-67

15. Tiikkainen M, Tamminen M, Hakkinen AM, Bergholm R, Vehkavaara S, Halavaara J, Teramo K, Rissanen A, Yki-Jarvinen H 2002 Liver-fat accumulation and insulin resistance in obese women with previous gestational diabetes. Obes Res 10:859-67

16. Kelley DE, Mandarino LJ 2000 Fuel selection in human skeletal muscle in insulin resistance: a reexamination. Diabetes 49:677-83

17. Bajaj M, Suraamornkul S, Piper P, Hardies LJ, Glass L, Cersosimo E, Pratipanawatr T, Miyazaki Y, DeFronzo RA 2004 Decreased plasma adiponectin concentrations are closely related to hepatic fat content and hepatic insulin resistance in pioglitazone-treated type 2 diabetic patients. J Clin Endocrinol Metab 89:200-6

18. Ryysy L, Hakkinen AM, Goto T, Vehkavaara S, Westerbacka J, Halavaara J, Yki-Jarvinen H 2000 Hepatic fat content and insulin action on free fatty acids and glucose metabolism rather than insulin absorption are associated with insulin requirements during insulin therapy in type 2 diabetic patients. Diabetes 49:749-58

19. Tiikkainen M, Bergholm R, Vehkavaara S, Rissanen A, Hakkinen AM, Tamminen M, Teramo K, Yki-Jarvinen H 2003 Effects of identical weight loss on body composition and features of insulin resistance in obese women with high and low liver fat content. Diabetes 52:701-7

20. Howald H, Boesch C, Kreis R, Matter S, Billeter R, Essen-Gustavsson B, Hoppeler H 2002 Content of intramyocellular lipids derived by electron microscopy, biochemical assays, and (1)H-MR spectroscopy. J Appl Physiol 92:2264-72

21. Thomas EL, Hamilton G, Patel N, O'Dwyer R, Dore CJ, Goldin RD, Bell JD, Taylor-Robinson SD 2005 Hepatic triglyceride content and its relation to body adiposity: a magnetic resonance imaging and proton magnetic resonance spectroscopy study. Gut 54:122-7

22. Tappy L, Paquot N, Tounian P, Schneiter P, Jequier E 1995 Assessment of glucose metabolism in humans with the simultaneous use of indirect calorimetry and tracer techniques. Clin Physiol 15:1-12

23. Ross R, Goodpaster B, Kelley D, Boada F 2000 Magnetic resonance imaging in human body composition research. From quantitative to qualitative tissue measurement. Ann N Y Acad Sci 904:12-7

24. Kamba M, Meshitsuka S, Iriguchi N, Koda M, Kimura K, Ogawa T 2000 Measurement of relative fat content by proton magnetic resonance spectroscopy using a clinical imager. J Magn Reson Imaging 11:330-5

25. Goodpaster BH 2002 Measuring body fat distribution and content in humans. Curr Opin Clin Nutr Metab Care 5:481-7

26. Ross R 2003 Advances in the application of imaging methods in applied and clinical physiology. Acta Diabetol 40 Suppl 1:S45-50

27. Taskinen MR, Packard CJ, Shepherd J 1990 Effect of insulin therapy on metabolic fate of apolipoprotein B- containing lipoproteins in NIDDM. Diabetes 39:1017-27.

28. Oscarsson J, Ottosson M, Eden S 1999 Effects of growth hormone on lipoprotein lipase and hepatic lipase. J Endocrinol Invest 22(5:2-9.

29. Carroll PV, Christ ER, Bengtsson BA, Carlsson L, Christiansen JS, Clemmons D, Hintz R, Ho K, Laron Z, Sizonenko P, Sonksen PH, Tanaka T, Thorne M 1998 Growth hormone deficiency in adulthood and the effects of growth hormone replacement: a review. Growth Hormone Research Society Scientific Committee. J Clin Endocrinol Metab 83:382-95.

30. Tomlinson JW, Holden N, Hills RK, Wheatley K, Clayton RN, Bates AS, Sheppard MC, Stewart PM 2001 Association between premature mortality and hypopituitarism. West Midlands Prospective Hypopituitary Study Group. Lancet 357:425-31.

31. Cameron AJ, Shaw JE, Zimmet PZ 2004 The metabolic syndrome: prevalence in worldwide populations. Endocrinol Metab Clin North Am 33:351-75, table of contents

32. Dunstan DW, Salmon J, Owen N, Armstrong T, Zimmet PZ, Welborn TA, Cameron AJ, Dwyer T, Jolley D, Shaw JE 2004 Physical activity and television viewing in relation to risk of undiagnosed abnormal glucose metabolism in adults. Diabetes Care 27:2603-9

33. Goodpaster BH, Kelley DE, Wing RR, Meier A, Thaete FL 1999 Effects of weight loss on regional fat distribution and insulin sensitivity in obesity. Diabetes 48:839-47

34. Doucet E, St-Pierre S, Almeras N, Imbeault P, Mauriege P, Pascot A, Despres JP, Tremblay A 2002 Reduction of visceral adipose tissue during weight loss. Eur J Clin Nutr 56:297-304

35. Decombaz J, Schmitt B, Ith M, Decarli B, Diem P, Kreis R, Hoppeler H, Boesch C 2001 Postexercise fat intake repletes intramyocellular lipids but no faster in trained than in sedentary subjects. Am J Physiol Regul Integr Comp Physiol 281:R760-9

36. Zehnder M, Christ ER, Ith M, Acheson K, Pouteau E, Kreis R, Trepp R, Diem P, Boesch C, Décombaz J 2005 Higher intramyocellular lipid (IMCL) concentrations lead to an increased energy contribution from IMCL and from total muscle fuel stores during exercise. submitted

37. Smekal G, von Duvillard SP, Pokan R, Tschan H, Baron R, Hofmann P, Wonisch M, Bachl N 2003 Effect of endurance training on muscle fat metabolism during prolonged exercise: agreements and disagreements. Nutrition 19:891-900

38. Larson-Meyer DE, Newcomer BR, Hunter GR 2002 Influence of endurance running and recovery diet on intramyocellular lipid content in women: a 1H NMR study. Am J Physiol Endocrinol Metab 282:E95-E106

39. Krssak M, Petersen KF, Bergeron R, Price T, Laurent D, Rothman DL, Roden M, Shulman GI 2000 Intramuscular glycogen and intramyocellular lipid utilization during prolonged exercise and recovery in man: a 13C and 1H nuclear magnetic resonance spectroscopy study. J Clin Endocrinol Metab 85:748-54

40. Roden M, Price TB, Perseghin G, Petersen KF, Rothman DL, Cline GW, Shulman GI 1996 Mechanism of free fatty acid-induced insulin resistance in humans. J Clin Invest 97:2859-65

41. Stein DT, Szczepaniak LS, Dobbins R, Malloy CR, JD. M 1997 Skeletal muscle triglyceride stores are increased in insulin resistance (Abstract). Diabetes 46 (Suppl.1):23A

42. Krssak M, Falk Petersen K, Dresner A, DiPietro L, Vogel SM, Rothman DL, Roden M, Shulman GI 1999 Intramyocellular lipid concentrations are correlated with insulin sensitivity in humans: a 1H NMR spectroscopy study. Diabetologia 42:113-6

43. Kautzky-Willer A, Krssak M, Winzer C, Pacini G, Tura A, Farhan S, Wagner O, Brabant G, Horn R, Stingl H, Schneider B, Waldhausl W, Roden M 2003 Increased intramyocellular lipid concentration identifies impaired glucose metabolism in women with previous gestational diabetes. Diabetes 52:244-51

44. Johnson NA, Stannard SR, Mehalski K, Trenell MI, Sachinwalla T, Thompson CH, Thompson MW 2003 Intramyocellular triacylglycerol in prolonged cycling with high- and low-carbohydrate availability. J Appl Physiol 94:1365-72

45. Gan SK, Kriketos AD, Poynten AM, Furler SM, Thompson CH, Kraegen EW, Campbell LV, Chisholm DJ 2003 Insulin action, regional fat, and myocyte lipid: altered relationships with increased adiposity. Obes Res 11:1295-305

46. Perseghin G, Scifo P, Danna M, Battezzati A, Benedini S, Meneghini E, Del Maschio A, Luzi L 2002 Normal insulin sensitivity and IMCL content in overweight humans are associated with higher fasting lipid oxidation. Am J Physiol Endocrinol Metab 283:E556-64

47. Thamer C, Machann J, Bachmann O, Haap M, Dahl D, Wietek B, Tschritter O, Niess A, Brechtel K, Fritsche A, Claussen C, Jacob S, Schick F, Haring HU, Stumvoll M 2003 Intramyocellular lipids: anthropometric determinants and relationships with maximal aerobic capacity and insulin sensitivity. J Clin Endocrinol Metab 88:1785-91

48. Stannard SR, Thompson MW, Fairbairn K, Huard B, Sachinwalla T, Thompson CH 2002 Fasting for 72 h increases intramyocellular lipid content in nondiabetic, physically fit men. Am J Physiol Endocrinol Metab 283:E1185-91

49. Seppala-Lindroos A, Vehkavaara S, Hakkinen AM, Goto T, Westerbacka J, Sovijarvi A, Halavaara J, Yki-Jarvinen H 2002 Fat accumulation in the liver is associated with defects in insulin suppression of glucose production and serum free fatty acids independent of obesity in normal men. J Clin Endocrinol Metab 87:3023-8

50. Tuomilehto J, Lindstrom J, Eriksson JG, Valle TT, Hamalainen H, Ilanne-Parikka P, Keinanen-Kiukaanniemi S, Laakso M, Louheranta A, Rastas M, Salminen V, Uusitupa M 2001 Prevention of type 2 diabetes mellitus by changes in lifestyle among subjects with impaired glucose tolerance. N Engl J Med 344:1343-50

51. Hanson RL, Imperatore G, Bennett PH, Knowler WC 2002 Components of the "metabolic syndrome" and incidence of type 2 diabetes. Diabetes 51:3120-7

52. Kopelman PG 1997 The effects of weight loss treatments on upper and lower body fat. Int J Obes Relat Metab Disord 21:619-25

53. Holt RI, Webb E, Pentecost C, Sonksen PH 2001 Aging and physical fitness are more important than obesity in determining exercise-induced generation of GH. J Clin Endocrinol Metab 86:5715-20

54. Jorgensen JO, Moller J, Alberti KG, Schmitz O, Christiansen JS, Orskov H, Moller N 1993 Marked effects of sustained low growth hormone (GH) levels on day-to- day fuel metabolism: studies in GH-deficient patients and healthy untreated subjects. J Clin Endocrinol Metab 77:1589-96.

55. White LJ, Ferguson MA, McCoy SC, Kim H 2003 Intramyocellular lipid changes in men and women during aerobic exercise: a (1)H-magnetic resonance spectroscopy study. J Clin Endocrinol Metab 88:5638-43

56. Kelley DE 2004 Influence of weight loss and physical activity interventions upon muscle lipid content in relation to insulin resistance. Curr Diab Rep 4:165-8

57. Schrauwen-Hinderling VB, Schrauwen P, Hesselink MK, van Engelshoven JM, Nicolay K, Saris WH, Kessels AG, Kooi ME 2003 The increase in intramyocellular lipid content is a very early response to training. J Clin Endocrinol Metab 88:1610-6

58. Christ ER, Cummings MH, Lumb PJ, Crook MA, Sonksen PH, Russell-Jones DL 1999 Growth hormone (GH) replacement therapy reduces serum sialic acid concentrations in adults with GH-deficiency: a double-blind placebo- controlled study. Clin Endocrinol (Oxf) 51:173-9.

59. Salomon F, Cuneo RC, Hesp R, Sonksen PH 1989 The effects of treatment with recombinant human growth hormone on body composition and metabolism in adults with growth hormone deficiency. N Engl J Med 321:1797-803.

60. Beshyah SA, Sharp PS, Gelding SV, Halliday D, Johnston DG 1993 Whole-body leucine turnover in adults on conventional treatment for hypopituitarism. Acta Endocrinol (Copenh) 129:158-64

61. Christ E, Cummings M, Russell-Jones D 1998 Dyslipidaemia in adult growth hormone deficiency and the effect of GH replacement therapy - a review. Trends in Endocrinology and Metabolism 9:200-206.

62. Jorgensen JO, Moller N, Wolthers T, Moller J, Grofte T, Vahl N, Fisker S, Orskov H, Christiansen JS 1995 Fuel metabolism in growth hormone-deficient adults. Metabolism 44:103-7.

63. Cuneo RC, Salomon F, Wiles CM, Hesp R, Sonksen PH 1991 Growth hormone treatment in growth hormone-deficient adults. II. Effects on exercise performance. J Appl Physiol 70:695-700.

64. Christ ER, Cummings MH, Westwood NB, Sawyer BM, Pearson TC, Sonksen PH, Russell-Jones DL 1997 The importance of growth hormone in the regulation of erythropoiesis, red cell mass, and plasma volume in adults with growth hormone deficiency. J Clin Endocrinol Metab 82:2985-90.

65. Trepp R, Boesch C, Ith M, Schmid J-P, Hoppeler H, Flück M, Diem P, Christ ER 2004 The effect of GH replacement therapy on exercise capacity, insulin resistance and intramyocellular lipids (IMCL) in patients with growth hormone deficiency. Poster presentation; International Congress of Endocrinology, ICE, Lisbon

66. Angeletti G 2003 The metabolic complications of acromegaly. J Endocrinol Invest 26:18-9

67. Nielsen S, Moller N, Christiansen JS, Jorgensen JO 2001 Pharmacological antilipolysis restores insulin sensitivity during growth hormone exposure. Diabetes 50:2301-8

68. Bengtsson BA, Eden S, Lonn L, Kvist H, Stokland A, Lindstedt G, Bosaeus I, Tolli J, Sjostrom L, Isaksson OG 1993 Treatment of adults with growth hormone (GH) deficiency with recombinant human GH. J Clin Endocrinol Metab 76:309-17.

69. Fowelin J, Attvall S, Lager I, Bengtsson BA 1993 Effects of treatment with recombinant human growth hormone on insulin sensitivity and glucose metabolism in adults with growth hormone deficiency. Metabolism 42:1443-7.

70. Twickler TB, Dallinga-Thie GM, Visseren FL, de Vries WR, Erkelens DW, Koppeschaar HP 2003 Induction of postprandial inflammatory response in adult onset growth hormone deficiency is related to plasma remnant-like particle-cholesterol concentration. J Clin Endocrinol Metab 88:1228-33

71. Cummings MH, Christ E, Umpleby AM, Albany E, Wierzbicki A, Lumb PJ, Sonksen PH, Russell-Jones DL 1997 Abnormalities of very low density lipoprotein apolipoprotein B-100 metabolism contribute to the dyslipidaemia of adult growth hormone deficiency. J Clin Endocrinol Metab 82:2010-3.

72. Kissebah AH, Alfarsi S, Evans DJ, Adams PW 1982 Integrated regulation of very low density lipoprotein triglyceride and apolipoprotein-B kinetics in non-insulin-dependent diabetes mellitus. Diabetes 31:217-25.

73. Dixon JL, Ginsberg HN 1992 Hepatic synthesis of lipoproteins and apolipoproteins. Semin Liver Dis 12:364-72.

74. Christ ER, Cummings MH, Albany E, Umpleby AM, Lumb PJ, Wierzbicki AS, Naoumova RP, Boroujerdi MA, Sonksen PH, Russell-Jones DL 1999 Effects of growth hormone (GH) replacement therapy on very low density lipoprotein apolipoprotein B100 kinetics in patients with adult GH deficiency: a stable isotope study. J Clin Endocrinol Metab 84:307-16.

75. Adams LA, Feldstein A, Lindor KD, Angulo P 2004 Nonalcoholic fatty liver disease among patients with hypothalamic and pituitary dysfunction. Hepatology 39:909-14

76. Sonksen PH, Christiansen JS 1998 Consensus guidelines for the diagnosis and treatment of adults with growth hormone deficiency. Growth Hormone Research Society. Growth Horm IGF Res 8 Suppl B:89-92.

77. Tappy L, Schneiter P 1997 Measurement of substrate oxidation in man. Diabetes Metab 23:435-42

78. Finegood DT, Bergman RN, Vranic M 1987 Estimation of endogenous glucose production during hyperinsulinemic-euglycemic glucose clamps. Comparison of unlabeled and labeled exogenous glucose infusates. Diabetes 36:914-24

79. Ferrannini E 1988 The theoretical bases of indirect calorimetry: a review. Metabolism 37:287-301

80. Robert JJ, Koziet J, Chauvet D, Darmaun D, Desjeux JF, Young VR 1987 Use of 13C-labeled glucose for estimating glucose oxidation: some design considerations. J Appl Physiol 63:1725-32

81. Tappy L 1995 Regulation of hepatic glucose production in healthy subjects and patients with non-insulin-dependent diabetes mellitus. Diabete Metab 21:233-40

82. Boesch C, Decombaz J, Slotboom J, Kreis R 1999 Observation of intramyocellular lipids by means of 1H magnetic resonance spectroscopy. Proc Nutr Soc 58:841-50

83. Schmitt B, Fluck M, Decombaz J, Kreis R, Boesch C, Wittwer M, Graber F, Vogt M, Howald H, Hoppeler H 2003 Transcriptional adaptations of lipid metabolism in tibialis anterior muscle of endurance-trained athletes. Physiol Genomics 15:148-57

84. Boesch C, Ith M, Jung B, Bruegger K, Erban S, Diamantis I, Kreis R, Bar A 2001 Effect of oral D-tagatose on liver volume and hepatic glycogen accumulation in healthy male volunteers. Regul Toxicol Pharmacol 33:257-67

85. Kamber M, Koster M, Kreis R, Walker G, Boesch C, Hoppeler H 1999 Creatine supplementation--part I: performance, clinical chemistry, and muscle volume. Med Sci Sports Exerc 31:1763-9

86. Fusch C, Slotboom J, Fuehrer U, Schumacher R, Keisker A, Zimmermann W, Moessinger A, Boesch C, Blum J 1999 Neonatal body composition: dual-energy X-ray absorptiometry, magnetic resonance imaging, and three-dimensional chemical shift imaging versus chemical analysis in piglets. Pediatr Res 46:465-73

87. Steele R 1959 Influences of glucose loading and of injected insulin on hepatic glucose output. Ann N Y Acad Sci 82:420-30

88. Bruce CR, Kriketos AD, Cooney GJ, Hawley JA 2004 Disassociation of muscle triglyceride content and insulin sensitivity after exercise training in patients with Type 2 diabetes. Diabetologia 47:23-30
